# World Health Organization classification of tumours of the breast 6th edition 2026

**DOI:** 10.1111/his.70149

**Published:** 2026-04-21

**Authors:** Cecily Quinn, Puay Hoon Tan, Kimberly H Allison, Edi Brogi, Sunil R Lakhani, Stuart J Schnitt, Stephen B Fox, Shabnam Jaffer, Aysegul Sahin, Roberto Salgado, Anna Sapino, Ernest Kwasi Adjei, Katherine Geiersbach, Thomas H Helbich, Matteo Lambertini, Christos Sotiriou, Jennelle C Hodge, Joseph D Khoury, Bharat Rekhi, Ales Ryska, Gary Tse, Andrew Field, Harshima Wijesinghe, Pavitratha Puspanathan, Christine Giesen, Blanca Iciar Indave Ruiz, Ian Ellis, Dilani Lokuhetty, WCT Standing Editorial Board, Wendy Cooper, Michael Eden, Andrew Field, Vicky Goh, Jennelle C. Hodge, James Kench, Joseph D. Khoury, Katia Leite, Zhiyong Liang, Daichi Maeda, George Netto, Bharat Rekhi, Miguel Reyes Mugica, Brian Rous, Ales Ryska, Shahin Sayed, Antonia Sepulveda, Chanjuan Shi, Gary Tse

**Affiliations:** ^1^ Department of Histopathology St. Vincent's University Hospital and University College Dublin Ireland; ^2^ Department of Pathology Luma Medical Centre, Singapore Singapore; ^3^ Department of Pathology Stanford University School of Medicine Stanford California USA; ^4^ Department of Pathology and Laboratory Medicine Memorial Sloan Kettering Cancer Center New York City New York USA; ^5^ Department of Pathology University of Queensland Centre for Clinical Research, Pathology Queensland and Sullivan Nicolaides Pathology Brisbane Queensland Australia; ^6^ Department of Pathology Beth Israel Deaconess Medical Center and Harvard Medical School Boston Massachusetts USA; ^7^ Department of Pathology Peter MacCallum Cancer Centre and Collaborative Centre Genomic Cancer Medicine, University of Melbourne Melbourne Victoria Australia; ^8^ Department of Pathology Northwell Health Lenox Hill Hospital New York City New York USA; ^9^ Department of Pathology The University of Texas MD Anderson Cancer Center Houston Texas USA; ^10^ Department of Pathology ZAS Hospitals Antwerpen Belgium; ^11^ Division of Research Peter MacCallum Cancer Centre Melbourne Victoria Australia; ^12^ Department of Pathology Candiolo Cancer Institute, FPO‐IRCCS Candiolo Torino Italy; ^13^ Department of Anatomic Pathology Komfo Anokye Teaching Hospital Kumasi Ghana; ^14^ Department of Laboratory Medicine and Pathology Mayo Clinic Rochester Minnesota USA; ^15^ Department of Biomedical Imaging and Image‐Guided Therapy, Division of General and Paediatric Radiology Medical University of Vienna/General Hospital Vienna Austria; ^16^ Department of Internal Medicine and Medical Specialties (DIMI), School of Medicine University of Genova Genoa Italy; ^17^ Academic Oncology Unit IRCCS Azienda Ospedaliera Metropolitana ‐ Plesso Galliera Genoa Italy; ^18^ Department of Medical Oncology Institut Jules Bordet Brussels Belgium; ^19^ Department of Medical and Molecular Genetics, and IU Simon Comprehensive Cancer Center Indiana University Indianapolis Indiana USA; ^20^ Department of Pathology, Microbiology, and Immunology University of Nebraska Medical Center Omaha Nebraska USA; ^21^ Department of Pathology Tata Memorial Centre, Homi Bhabha National Institute (HBNI) University Mumbai India; ^22^ The Fingerland Department of Pathology University Hospital Hradec Kralove Hradec Kralove Czechia; ^23^ Department of Anatomical and Cellular Pathology The Chinese University of Hong Kong Hong Kong SAR China; ^24^ Department of Anatomical Pathology St Vincent's Hospital Sydney New South Wales Australia; ^25^ University of NSW Sydney Sydney New South Wales Australia; ^26^ University of Notre Dame Sydney New South Wales Australia; ^27^ International Agency for Research on Cancer, World Health Organization Lyon France; ^28^ Department of Histopathology Nottingham University Hospitals Nottingham UK

**Keywords:** 6th edition, breast tumour classification, WHO classification of tumours

## Abstract

The 6th edition of the WHO Classification of Breast Tumours introduces both major and minor changes based on recent advances in our understanding of breast biology, developments in diagnostic modalities, identification of specific molecular targets and new treatment regimens necessitating modifications to pathology reporting and tumour biomarker categorisation. This review summarises the main changes that strive towards a classification of global relevance. In invasive carcinoma, predictive factors increasingly inform modern breast cancer treatment. The 6th edition provides an update on HER2 reporting categories following the DESTINY‐Breast 04 and 06 trials. Terminologies used to classify invasive tumours are clarified, with the term ‘variant’ now reserved for molecular/genetic alterations. Invasive lobular carcinoma (ILC) with extracellular mucin is recognised as a new diagnostic entity with prognostic implications. The diagnosis of mucinous carcinoma (MC) is reserved for mucin secreting carcinomas with grade 1 or 2 morphology and a favourable biomarker profile. The diagnosis of malignant phyllodes tumours requires only four of the original five adverse histological criteria. Classification of neuroendocrine tumours (NETs) is revised, recognising that the unified model, promoted in the 5th edition, is difficult to apply to the breast. New approaches to the classification of adenomyoepithelioma are discussed but the 5th edition system is broadly retained. A new section on ‘Small Diagnostic Samples’ outlines the merits of non‐operative biopsy diagnosis, the B coding system and the importance of multidisciplinary review. Changes to diagnostic practice and the emerging role of artificial intelligence, with advantages and challenges, are discussed in a new section on ‘Digital Pathology’.

## Graphical abstract

The 6th edition of the World Health Organization Classification of Breast Tumours introduces changes based on recent advances in our understanding of breast biology, developments in diagnostic modalities, identification of specific molecular targets and new treatment regimes. This review summarises the main changes in the 6th edition, with emphasis on accurate diagnosis and clinical implications.

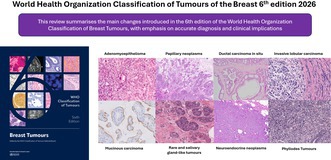

**Abbreviations**
ADC: antibody drug conjugate
AME: adenomyoepithelioma
CAP: College of American Pathologists
CNB: core needle biopsy
ctDNA: circulating tumour DNA
DCIS: ductal carcinoma *in situ*
EMA: epithelial membrane antigen
EPC: encapsulated papillary carcinoma
FNAB: fine needle aspiration biopsy
HPFs: high power fields
IARC: International Agency for Research on Cancer
IBC: invasive breast carcinoma
ICCR: International Collaboration on Cancer Reporting
IHC: immunohistochemistry
ILC: Invasive lobular carcinoma
ILCEM: Invasive lobular carcinoma with extracellular mucin
ILC TE: Invasive lobular carcinoma with tubular elements
M‐AME: malignant AME
MC: mucinous carcinoma
NEC: neuroendocrine carcinoma
NEN: neuroendocrine neoplasm
NETs: neuroendocrine tumours
NST: no special type
PASH: pseudoangiomatous stromal hyperplasia
SCNEC: small cell neuroendocrine carcinoma
SPC: solid papillary carcinoma
T‐DXd: trastuzumab deruxtecan
VAB: vacuum assisted biopsy
WHO: World Health Organization

## Introduction

The World Health Organization (WHO) classification of breast tumours provides a uniform nomenclature and standardised framework for categorising breast tumours, facilitating accurate diagnosis, guiding therapeutic strategies, informing breast cancer research and enabling accurate epidemiological data collection by cancer registries. The 6th edition of the WHO Classification of breast tumours encompasses both major and minor changes from the 5th edition, and incorporates new photomicrographs and digital images. The inclusion on the editorial board of oncology, radiology and molecular genetics experts as well as pathologists practicing in low‐ and middle‐income country settings has strengthened the increasingly multidisciplinary nature of the classification while ensuring its global relevance. The process of developing the classification was supported by the guidance and suggestions provided by the newly formed WHO Classification of tumours subcommittees^1^ and the outcomes of the European Union funded WCT EVI MAP project.^2^, ^3^

## New Sections

### Small Diagnostic Samples

The new section on *small diagnostic samples* profiles the current sampling techniques used for non‐operative breast diagnosis [fine needle aspiration biopsy (FNAB), core needle biopsy (CNB) and vacuum assisted biopsy (VAB)], the corresponding imaging modalities employed and the optimal techniques for evaluation of different imaging abnormalities. The importance of standardised reporting, with the use of a classification system (e.g., the *B coding system* for categorising breast biopsy findings) (UK non‐operative guidelines)^4^ and the role of triple assessment, with correlation of pathological, clinical and imaging findings, are emphasised. While FNAB is less commonly used in contemporary clinical practice in high income countries, it retains an important role in the evaluation of breast lesions particularly in many resource constrained settings. The current development of a WHO reporting system for breast cytopathology as a collaborative project of WHO—International Agency for Research on Cancer with the International Academy of Cytology, which is in progress, will assist in standardising FNAB reporting and clinical usefulness.

### Digital pathology

Digital pathology, encompassed under the broad umbrella term of computational pathology, including artificial intelligence (AI) applications, is being progressively integrated into routine laboratory workflows, enabling remote diagnostic reporting, convenient retrieval of digitally stored archival cases for teaching, review and multidisciplinary tumour board discussions, and for research.

Digital pathology offers additional advantages as AI tools and algorithms enhance and augment pathologist assessment. Many useful AI algorithms have progressed beyond their initial research phase and are now commercially available, some of which aid in tasks such as biomarker stain quality control and interpretation, tissue/tumour detection, grading of cancers, including mitoses counting, and identification of specific findings such as invasive cancer, ductal carcinoma *in situ*, microcalcifications and lymph node metastases.^5^, ^6^ Work is ongoing to fine‐tune AI algorithms to assess tumour infiltrating lymphocytes,^7^ lymphovascular invasion and other features. The hope is that these tools will help to standardise practice and increase pathologist efficiency; however, some limitations and barriers remain and currently no single tool can aid with all elements of a breast pathology report.

More complex tasks leveraging digital pathology, using advanced computational pathology tools,^8^ reaffirm known genotype–phenotype associations^9^, ^10^ and elucidate novel prognostic features.^11^, ^12^ Studies report the ability of AI models to predict underlying driver gene mutations,^13^, ^14^, ^15^, ^16^, ^17^ tumour mutation burden,^18^ and the presence of homologous recombination deficiency.^19^, ^20^ Some AI tools can assist breast cancer subtyping^21^, ^22^, ^23^ and predict some treatment responses.^24^, ^25^, ^26^

Despite implementation challenges beyond the required infrastructure (including establishment costs for IT platforms, space, staff, reimbursement, analytical and clinical validation, scientific and regulatory hurdles, accountability, trust, economic impact, and environmental effects) digital pathology and AI are expected to be increasingly integral to breast pathology practice, especially in validating AI‐assisted breast cancer diagnosis, warranting mention in the 6th edition.^27^

## Updates

### Epithelial Tumours of the Breast

In the 6th edition, epithelial tumours of the breast are organised into: Part 1 which includes benign epithelial proliferations and precursors, adenosis and sclerosing lesions, adenomas, epithelial–myoepithelial tumours, papillary neoplasms, non‐invasive lobular neoplasia and ductal carcinoma in situ; and Part 2 which includes sections on prognosis and prediction, molecular pathology, invasive breast carcinoma (IBC) types, microinvasive carcinoma, rare and salivary gland‐type tumours, and NETs.

## Part 1

### Adenomyoepithelioma

The classification of adenomyoepithelioma (AME) poses diagnostic difficulty for pathologists, requiring a balance between recognition of potentially biologically aggressive forms and the risk of over‐treatment. The 6th edition editorial board considered the recent proposal for a modified classification of AME based on morphological characteristics aiming to refine treatment algorithms.^28^ The board concluded that there was insufficient evidence on patient outcomes to adopt this classification system at this time and the classification scheme of the 5th edition is retained.

**Figure 1:**
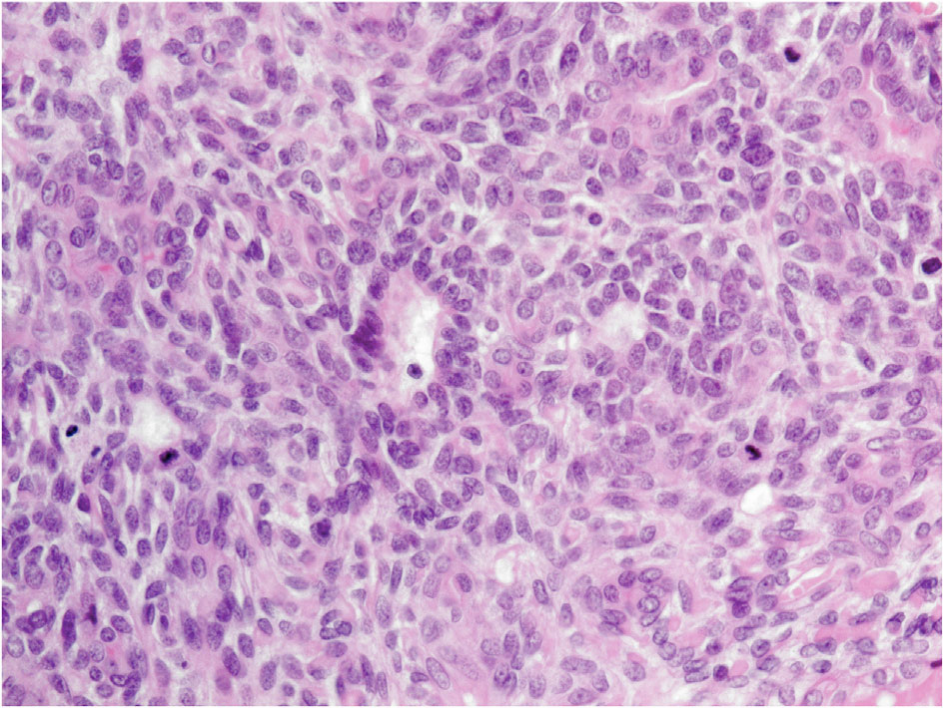
Atypical adenomyoepithelioma (case courtesy of Professor Emad Rakha). Adenomyoepithelioma (AME) with increased mitotic activity in the epithelial and myoepithelial cell components.

Classic AME incorporates benign and atypical forms, and presents a range of morphological patterns including tubular, spindle cell, lobulated and papillary. Furthermore, classic tubular AME may display an infiltrative outline, sometimes referred to as adenomyoepithelial adenosis. Mitotic activity may be observed in either or both the epithelial and myoepithelial components of classic AME with up to 1.5 mitoses per mm^2^ (3 mitoses per 10 high power fields (HPFs) of 0.5 mm in diameter and 0.2 mm^2^ in area) permissible. Criteria for designation as atypical AME (Figure 1) include the presence of mild to moderate cytologic atypia in either the epithelial or myoepithelial cell components with up to 2.5 mitoses per mm^2^ (5 mitoses per 10 HPFs of 0.5 mm in diameter and 0.2 mm^2^ in area).^28^ The presence of atypical mitotic figures should raise suspicion for malignant transformation. Focal necrosis can be observed in atypical AME. In occasional AMEs, the extent and degree of epithelial cell atypia may justify a diagnosis of ductal carcinoma *in situ* (DCIS) arising in an AME, which should be reported and managed in accordance with DCIS management protocols.^29^, ^30^

**Figure 2:**
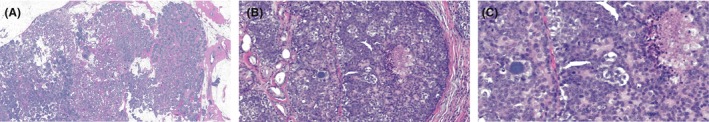
Malignant AME (case courtesy of Dr Young Kyung Bae). (**A**) Tubular adenomyoepithelioma with an adenomyoepithelial adenosis pattern shows bilayered tubules extending irregularly into the surrounding breast tissue. (**B**) Expanded biphasic solid epithelial islands show necrosis, nuclear atypia, and many mitoses exceeding 10 per 10 high power fields. (**C**) Higher magnification reveals several mitoses per high power field.

**Figure 3:**
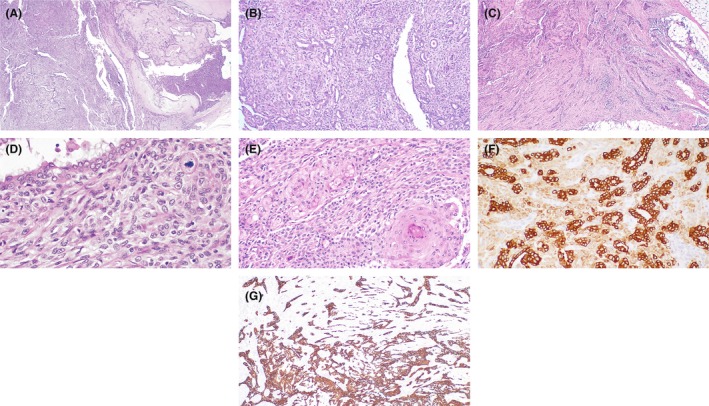
AME with metaplastic carcinoma. (**A**) Papillary adenomyoepithelioma shows anastomosed fronds covered by bilayered epithelial and myoepithelial cells with areas of hyalinised stroma. (**B**) Higher magnification shows prominent myoepithelial cells swirling around epithelial tubules. (**C**) The periphery of the papillary adenomyoepithelioma shows irregular epithelial nests and cords permeating into surrounding stroma. (**D**) Nuclear atypia and mitoses are present in ovoid to elongated, spindled myoepithelial cells. (**E**) Squamous differentiation is seen in the spindled component of the tumour. (**F**) CK7 immunohistochemistry highlights the epithelial cells of tubules and small nests. (**G**) CK14 immunohistochemistry reveals an intricately anastomosing pattern of invasive carcinoma. [Colour figure can be viewed at wileyonlinelibrary.com]

In the 6th edition, malignant AME (M‐AME) is defined as an AME with carcinoma which may arise from the epithelial cells, myoepithelial cells or, in some tumours, from both cell types. The underlying morphological features of an AME are identifiable with transition to carcinoma observed as cytological atypia, increased mitoses, and necrosis in the malignant component^31^, ^32^ (Figure 2). In M‐AME with malignant transformation of the epithelial component, the invasive carcinoma may be of no special type (NST) or a special type including lobular carcinoma.^31^, ^33^ In M‐AME with malignant transformation of the myoepithelial component, the carcinoma usually displays features of metaplastic spindle cell carcinoma^31^, ^33^, ^34^ (Figure 3). Other tumour types reported in M‐AME include metaplastic carcinoma with squamous cell carcinoma, low‐grade adenosquamous, and matrix‐production/heterologous mesenchymal differentiation^35^ as well as adenoid cystic carcinoma.^36^, ^37^ Immunohistochemical studies may be used to highlight the biphasic epithelial–myoepithelial nature of these tumours. Importantly, M‐AME may exhibit an aberrant immunoprofile, with loss of clear distinction between the dual cell populations.^28^ Immunohistochemical detection of the Q61R mutant isoform of *HRAS* is moderately sensitive but highly specific and may be used to support a diagnosis of an AME.^38^

In rare cases, malignancy can affect both the epithelial and myoepithelial cell components giving rise to biphasic carcinomas. The individual components should be described in the pathology report and the use of the term *epithelial myoepithelial carcinoma* to describe these tumours is discouraged.

### Papillary Neoplasms

**Figure 4:**
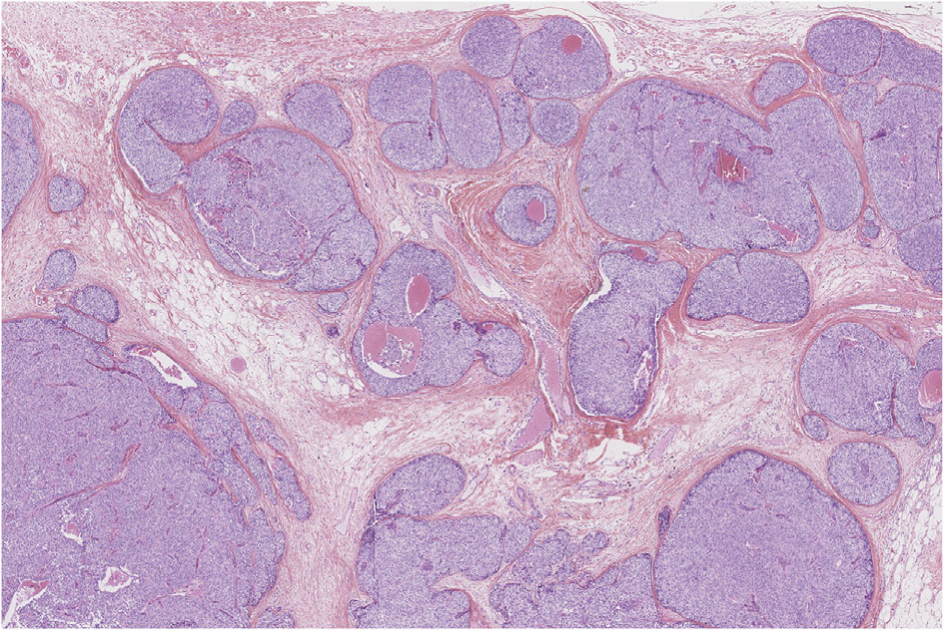
Solid papillary carcinoma (SPC) in situ. Expansile nodules with a solid growth pattern have inconspicuous, delicate fibrovascular cores, with no convincing evidence of invasion. [Colour figure can be viewed at wileyonlinelibrary.com]

**Figure 5:**
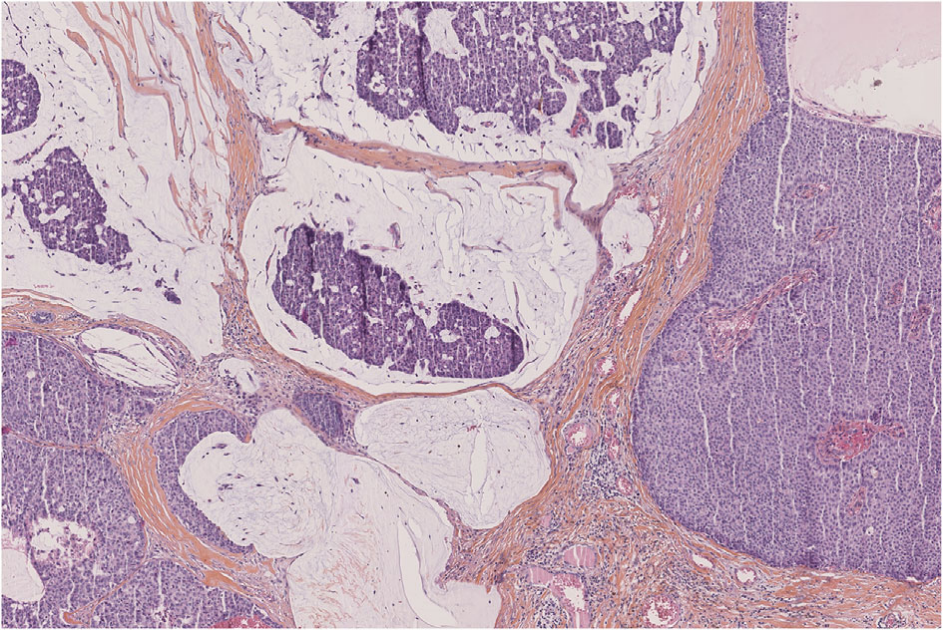
SPC with invasion with mucinous carcinoma. The invasive carcinoma is a mucinous carcinoma comprising rounded solid nodules in association with large clusters of tumour cells within pools of extracellular mucin. [Colour figure can be viewed at wileyonlinelibrary.com]

**Figure 6:**
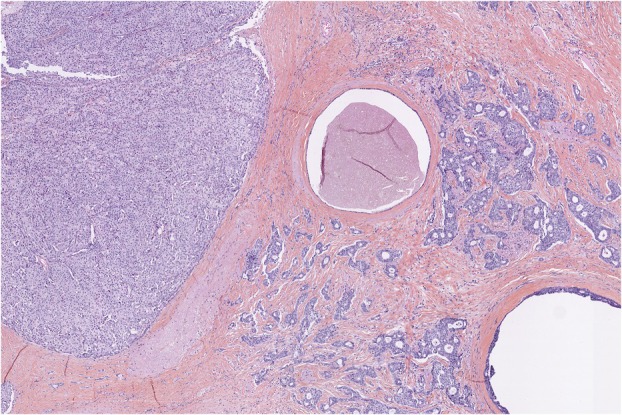
SPC in situ with invasive carcinoma. The invasive carcinoma is an invasive carcinoma of no special type (NST) comprising irregular neoplastic glands and trabeculae that infiltrate the breast parenchyma adjacent to solid papillary carcinoma. [Colour figure can be viewed at wileyonlinelibrary.com]

**Figure 7:**
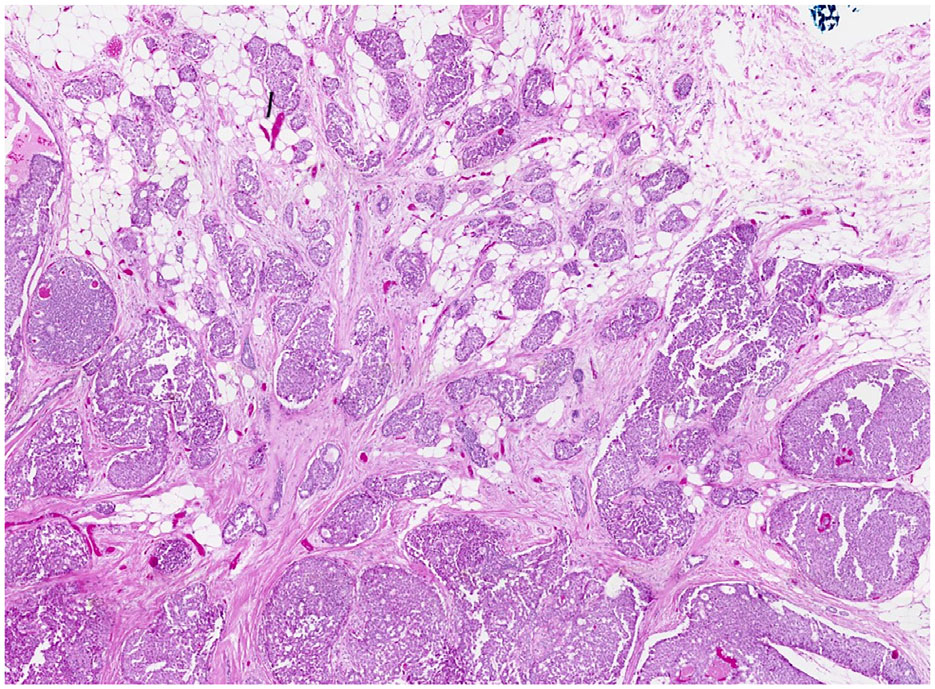
Invasive solid papillary carcinoma. Tumour nodules devoid of myoepithelium with ragged contours creating a geographical jigsaw pattern within a desmoplastic stroma. The cells have a neuroendocrine morphology. [Colour figure can be viewed at wileyonlinelibrary.com]

Key changes in the 6th edition include the clarification of invasive solid papillary carcinoma (SPC) into SPC *in situ* (Figure 4) with invasion and invasive SPC. SPC *in situ* with invasion designates SPC *in situ* associated with conventional invasive carcinoma, usually hypercellular MC (Figure 5) or invasive breast carcinoma NST (Figure 6), although other invasive carcinoma types can be encountered. In comparison, invasive SPC (Figure 7) consists of tumour nodules with solid papillary morphology invested by fine vessels, devoid of peripheral myoepithelial rims, with ragged and jagged contours creating a geographical jigsaw pattern within desmoplastic stroma. SPC composed of circumscribed nodules with smooth contours is classified as in situ disease, even in the absence of myoepithelial cells.

**Figure 8:**
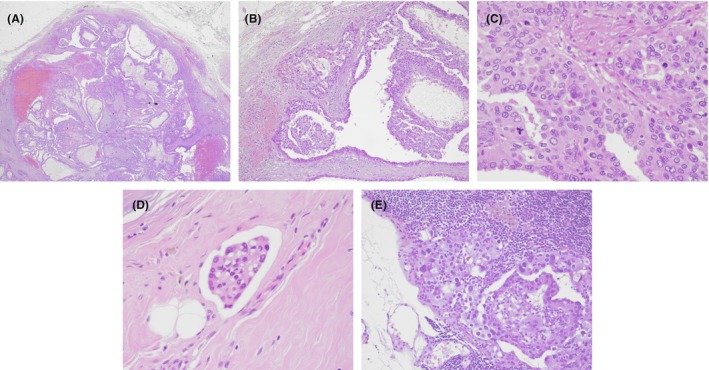
Encapsulated papillary carcinoma (EPC)‐like invasive carcinoma. (**A**) Low‐power view showing a well‐developed papillary configuration resembling EPC. (**B**) Focally irregular tumour outline. (**C**) High magnification showing marked cytological atypia and atypical mitotic figures. (**D**) Focus of lymphovascular invasion in the vicinity of the tumour. (E) Ipsilateral axillary lymph node containing a metastasis. [Colour figure can be viewed at wileyonlinelibrary.com]

An additional update relates to a very uncommon scenario that can be encountered in encapsulated papillary carcinoma (EPC) cases. In the 5th edition, the diagnosis of EPC (which is staged as a form of DCIS despite a frequent lack of peripheral myoepithelial markers) was reserved for cases with classic low‐intermediate grade and ER positive characteristics. It was recommended that EPC with high nuclear grade, increased mitotic activity and/or a triple negative or HER2 positive phenotype be graded, staged and managed as invasive disease. This was recommended to avoid the misclassification of high‐grade forms of invasive cancers that have pushing borders and pseudopapillary areas that can mimic EPC‐like architecture (Figure 8). However, the 6th edition acknowledges that there may be very rare forms of EPC with uncharacteristic atypical features (such as high‐grade features or ER negativity) that may still be appropriately categorised as forms of EPC.^39^, ^40^ The challenge of this distinction is particularly relevant on core biopsy when neoadjuvant treatment may be considered. Thorough histologic examination of the surgical excision specimen, potential expert consultation, clinical correlation and multidisciplinary discussion may be needed in challenging cases. A note or comment enumerating the unusual features and the limited data on their clinical implications is advised if this rare scenario is encountered. The vast majority of EPCs are ER positive and low‐intermediate nuclear grade.

### Ductal Carcinoma In Situ

**Figure 9:**
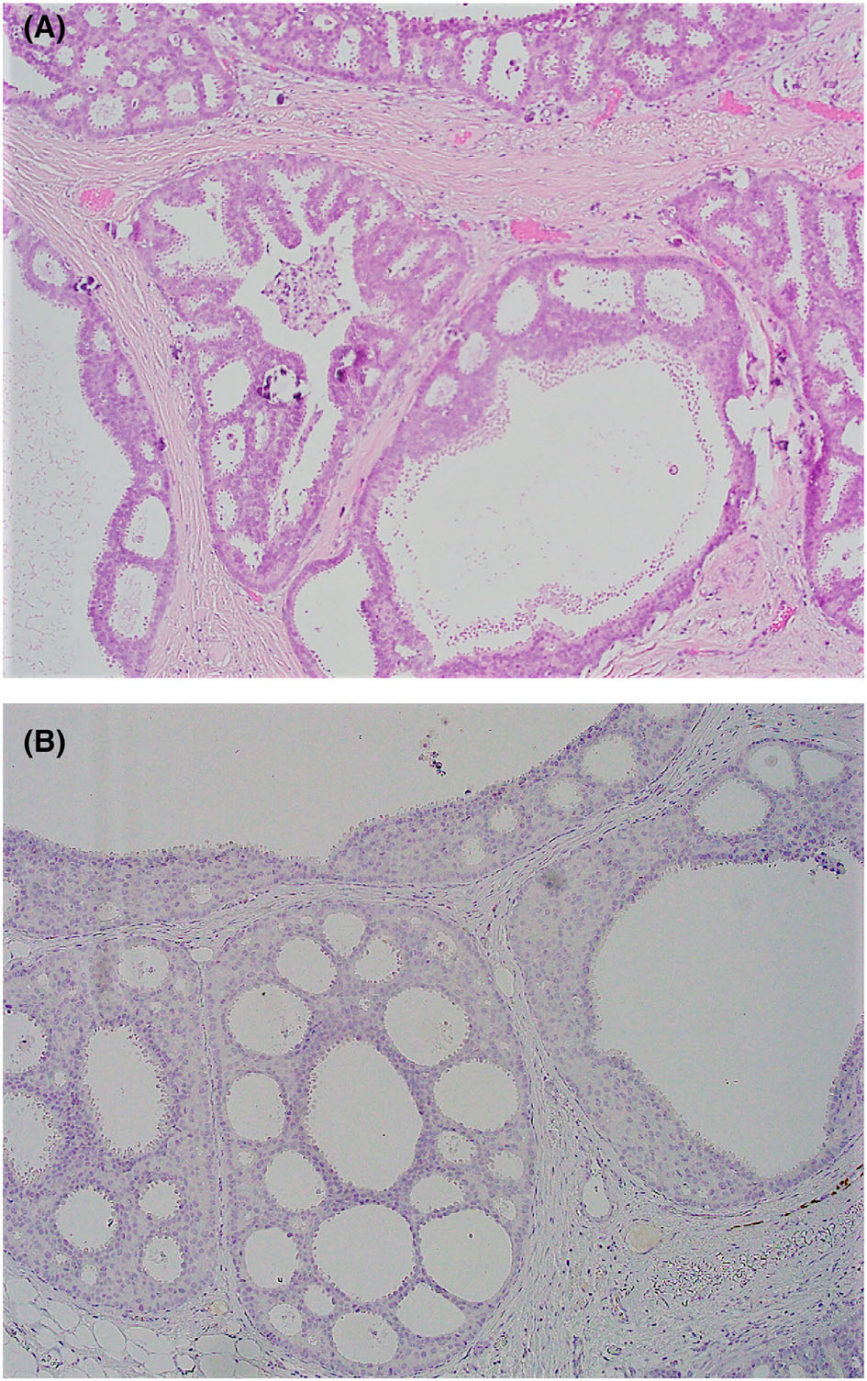
Ductal carcinoma in situ (DCIS) with no discernible peripheral myoepithelial cell layer, but retaining the architectural configuration of DCIS. (**A**) H&E shows cribriform DCIS morphology with focal necrosis and calcifications. (**B**) p63 immunohistochemistry shows absence of a myoepithelial rim around ducts with DCIS morphology (note internal control). [Colour figure can be viewed at wileyonlinelibrary.com]

The distinction of DCIS involving sclerosing lesions from invasive carcinoma remains challenging and the 6th edition reinforces the importance of careful examination for small irregular abnormal cell clusters that may represent microinvasion, especially when there is a prominent lymphocytic infiltrate. The role, limitations and use of more than one myoepithelial cell marker are reiterated. Additionally, the difficulty of classifying lesions that have the histologic appearance of DCIS but in which no myoepithelial cells can be demonstrated around tumour cell nests (Figure 9) is highlighted as an unresolved issue in the 6th edition. While the presence of surrounding basement membrane by immunohistochemistry (IHC) may be used to support the diagnosis of DCIS, it is advocated that the lack of both myoepithelial cell markers and demonstrable basement membrane should not result in a diagnosis of invasive carcinoma in the absence of a recognisable pattern of invasive carcinoma, similar to recommendations provided for encapsulated and solid papillary carcinomas.

**Figure 10:**
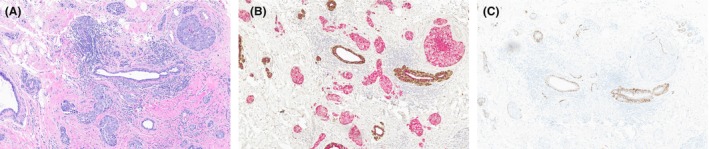
Occlusive lymphovascular invasion mimicking DCIS. (**A**) H&E shows solid tumour nests with neat outlines, reminiscent of DCIS. (**B**) ADH5 (CK 5/14, p63 and CK7/18) shows absence of myoepithelial cells around the solid tumour nests, accompanied by small groups of invasive carcinoma. (**C**) D2‐40 reveals partial rimming of the solid tumour nests, in keeping with lymphatic endothelial cells (although D2‐40 also decorates myoepithelial cells, the negative ADH5 immunostain suggests that the positive staining here represents lymphatic endothelial cells). [Colour figure can be viewed at wileyonlinelibrary.com]

Another scenario mentioned in this chapter is the resemblance to DCIS of large tumour emboli occluding and distending lymphovascular spaces. In contrast to DCIS, tumour emboli are haphazardly distributed, are devoid of myoepithelium and basement membrane, and no inflammation or reactive changes are present in the surrounding stroma. Immunohistochemical stains for myoepithelial markers are negative, whereas endothelial markers highlight residual endothelial cells lining the lymphovascular spaces, though D2‐40, a lymphatic endothelial cell marker, also decorates myoepithelial cells and may not be discriminatory (Figure 10).^41^

## Part 2

### Classification of Invasive Carcinoma

#### General overview: molecular classification, prognosis and prediction

Invasive breast cancers continue to be classified in the 6th edition based on morphologically defined types. However, prognostic and predictive factors as well as molecularly defined classification schemes have impacted much of our modern breast cancer treatment paradigms. The 5th edition included a large ‘General overview’ chapter to cover these important topics. In the 6th edition these are separated into several parts including an introduction, molecular classification and prognosis and prediction sections.

**Figure 11:**
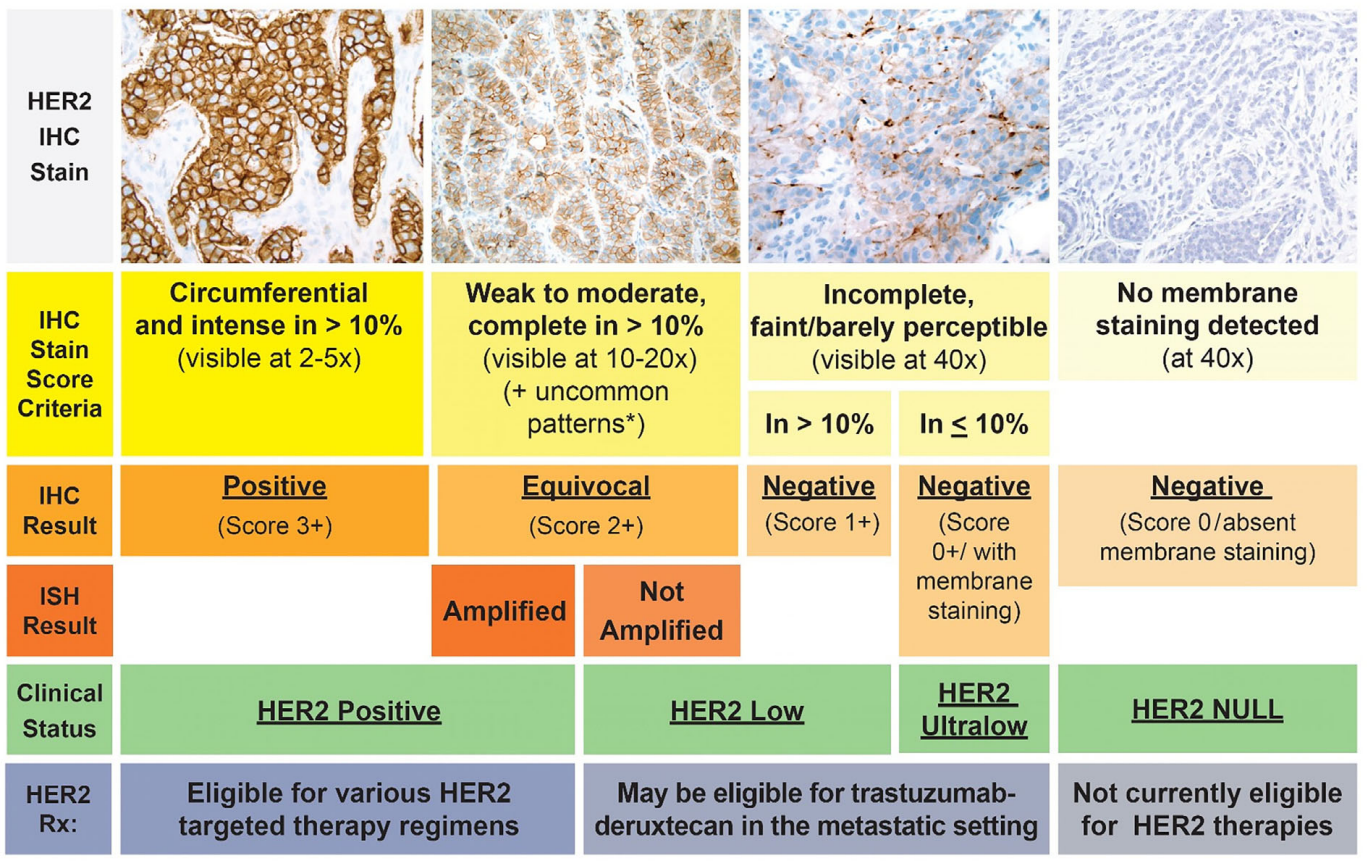
ERBB2 (HER2) interpretation and clinical implications. HER2 immunohistochemistry and in situ hybridisation results and their clinical implications for overall HER2 status and potential treatment options. IHC, immunohistochemistry; ISH, in situ hybridisation; Rx, treatment. [Colour figure can be viewed at wileyonlinelibrary.com]

Probably the most clinically relevant update in invasive breast cancer biomarkers since the last edition is related to HER2 testing reporting recommendations. The DESTINY‐Breast 04 and 06 trials have changed the clinical relevance of lower levels of HER2 expression detected by IHC. Thus, it is now important to pay attention to the discrimination of cases with no HER2 expression at all (0/absent membrane staining) from cases with any membrane staining to identify patients with metastatic disease eligible for treatment with the antibody drug conjugate (ADC), trastuzumab deruxtecan (T‐DXd).^42^, ^43^ Current testing guidelines and consensus statements on HER2 low breast cancers address best practices to aid in recognition of these subtle low level staining differences and reporting standards.^44^, ^45^, ^46^ Details of the semi‐quantitative IHC scores, including specifying the specific staining pattern in IHC 0 cases (e.g., 0/absent membrane staining vs 0+/with membrane staining), is recommended but reporting using HER2 low and ultra‐low terminology is not required (Figure 11).

New developments in breast cancer biomarkers, in particular ER positive metastatic cancers, include the relevance of circulating tumour DNA (ctDNA) in plasma (‘liquid biopsy’) to detect targetable mutations (such as *PIK3CA*, *PTEN* and *AKT*) or to identify the emergence of *ESR1* mutations that indicate resistance to aromatase inhibitors and prompt therapy changes.^47^, ^48^, ^49^, ^50^, ^51^, ^52^ However, ctDNA sensitivity is influenced by tumour shedding and tumour fraction, and a negative liquid biopsy does not exclude clinically relevant alterations, supporting the ongoing value of tissue‐based genomic profiling, particularly when ctDNA is negative/low fraction or when copy‐number–type events are of interest.^53^, ^54^ Evidence is evolving about the additional clinical applications of circulating biomarkers for early detection of cancer or of relapse, staging and monitoring patients with localised or advanced cancer, monitoring the efficacy of therapies, tracking tumour evolution and identifying resistance mechanisms.^55^, ^56^, ^57^

Standardisation of the assessment of post‐treatment response using protocols like those applied in the Residual Cancer Burden grossing and microscopic examination guidelines is also now recommended. The International Collaboration on Cancer Reporting (ICCR), College of American Pathologists (CAP), and other consortia have standardised reporting recommendations for postneoadjuvant breast cancer specimens.^58^, ^59^, ^60^

### Terminology

Terminology used to classify invasive tumours is clarified. A *subtype* refers to a tumour differing from the main tumour type in at least one dimension (clinical, histological or genetic), preferably resulting in a different treatment or outcome. A *pattern* differs from the main tumour type in morphological aspects only, but not in underlying biological or molecular features, treatment or patient outcome. The use of the term *'variant'* to describe differences in tumour characteristics is discouraged and should be reserved for molecular/genetic alterations.

### Invasive Lobular Carcinoma

**Figure 12:**
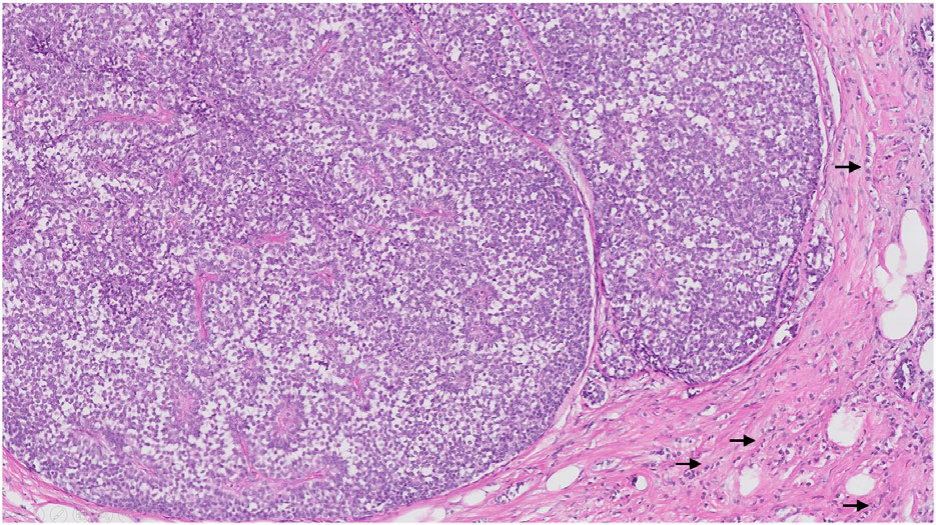
Solid papillary invasive lobular carcinoma shows expansile, well‐delimited tumour nodules composed of a discohesive cell population arranged in a solid papillary architecture, with linear cords of invasive lobular carcinoma in the adjacent stroma (arrows point to several of these linear tumour cords). [Colour figure can be viewed at wileyonlinelibrary.com]

ILC, which is classified as a breast cancer type, includes ILC, classic, and other forms. In this update, *special architectural patterns* (solid, alveolar, and trabecular) and *special cytological features* (signet ring cell, apocrine, histiocytoid, and pleomorphic) are described in more detail, noting that overlapping architectural and cytological patterns may be observed. The *trabecular pattern* may resemble invasive breast carcinoma (IBC) of no special type (IBC‐NST) and is likely under‐recognised. *Solid papillary ILC* is a variation of the solid pattern that displays a solid papillary architecture and appears well delimited with a pseudocapsule and is often associated with satellite foci of ILC at the periphery (Figure 12). Awareness of this entity avoids confusion with other papillary lesions, particularly in small biopsy specimens.

**Figure 13:**
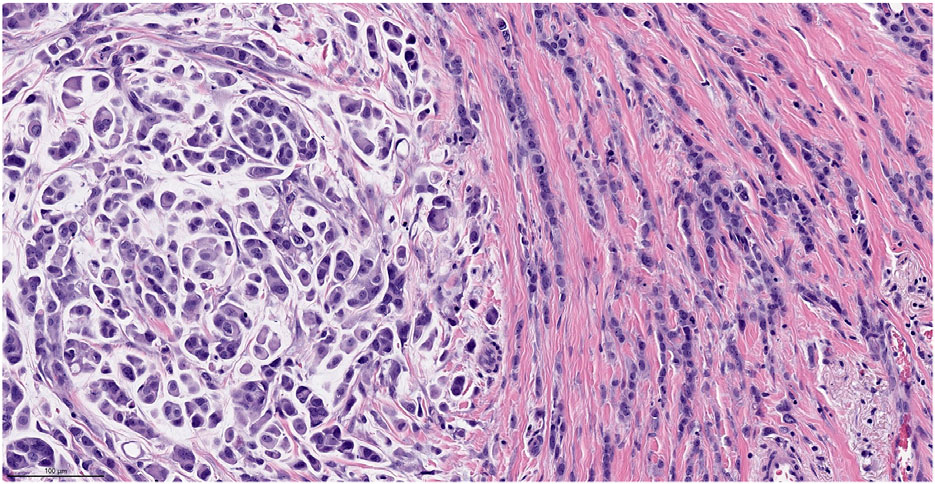
Invasive lobular carcinoma with extracellular mucin. A discohesive growth pattern and pleomorphic tumour cells are seen. Some of the tumour cells are suspended in extracellular mucin (left). A non‐mucinous component is usually present (right), as shown in this case. [Colour figure can be viewed at wileyonlinelibrary.com]

*Invasive lobular carcinoma with extracellular mucin* (ILCEM) (Figure 13) is a recently described emerging ILC subtype.^61^ This should be distinguished from classic ILC and MC in view of its more aggressive biological behaviour and less favourable prognosis.

**Figure 14:**
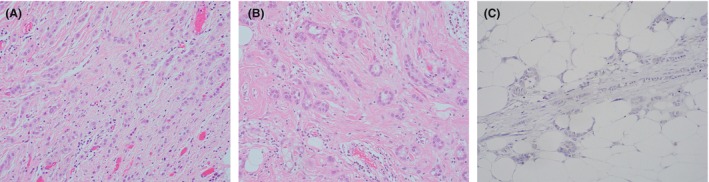
Invasive lobular carcinoma with tubule formation. (**A**) Classical invasive lobular carcinoma in part of the tumour. (**B**) Other areas showing similar cytological features with distinct tubule formation. (**C**) Both elements of the tumour are E‐cadherin negative. [Colour figure can be viewed at wileyonlinelibrary.com]

A recent proposal to classify tumours with mixed tubular and lobular elements that lack e‐cadherin expression (with p‐cadherin expression in the tubular component) as *invasive lobular carcinoma with tubular elements* (ILC TE)^62^ was not endorsed in the 6th edition due to insufficient evidence to merit a change in terminology at this time. At present, it is recommended that tumours with this morphological and immunohistochemical profile are regarded as true tubulo‐lobular carcinomas, and regarded as ILC with a very good prognosis^63^, ^64^ (Figure 14). Tumours exhibiting E‐cadherin negative ILC elements with E‐cadherin positive tubular elements are classified as mixed ILC and tubular carcinomas.

Some ILCs may show aberrant E‐cadherin expression or uncommonly have membranous E‐cadherin retention but loss of membrane staining of other proteins in the cadherin‐catenin complex such as p120 and β‐catenin.^65^, ^66^, ^67^, ^68^, ^69^, ^70^, ^71^, ^72^, ^73^, ^74^, ^75^ In the IBC‐NST chapter, it is noted that some breast cancers have indeterminate features such that the definite distinction between IBC‐NST and ILC is not clear despite the use of IHC. The 6th edition recommends calling these IBC‐NST with ‘lobular features/lobular growth pattern’. While there are differences in long‐term outcomes and patterns of spread, currently there are no clinical major treatment differences in the treatment of IBC‐NST vs ILC, with treatment pathways focused on biomarkers, predictive assays, stage and grade at diagnosis. However, increasing knowledge of the biology of ILC may lead to further refinement of the classification system and potentially clinical treatment differences in the coming years.

### Mucinous Carcinoma with Micropapillary Morphology

The importance of using strict criteria for the favourable histology type, pure MC, is emphasised with particular attention to excluding high‐grade forms of mucin‐producing cancer from this diagnosis.

**Figure 15:**
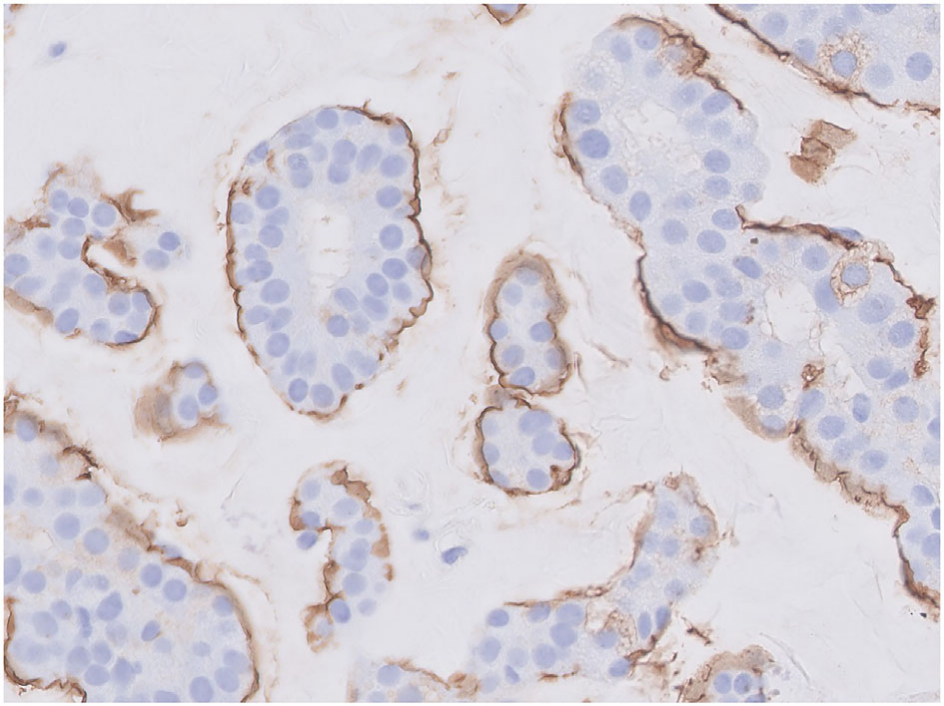
Mucinous carcinoma. Peripheral EMA staining is often seen in mucinous carcinoma (this finding alone is not considered diagnostic of micropapillary differentiation). [Colour figure can be viewed at wileyonlinelibrary.com]

The evidence to define a specific micropapillary pattern of MC is also reviewed. Since both classic forms of pure MC and those with more prominent micropapillary morphology frequently have peripheral epithelial membrane antigen (EMA) (Figure 15) and mucin 1 (MUC1) expression,^76^ IHC staining pattern is not useful as a distinguishing characteristic. The literature describing MCs with prominent micropapillary morphology associate this morphology with a younger age at diagnosis, higher incidence of lymph node metastases and a worse prognosis,^77^, ^78^ but these differences may be related to the higher nuclear grade^79^ and HER2 positivity, also described in the cancers included in these studies.^80^ When high nuclear grade and HER2 positive mucin‐producing cancers are excluded, it appears that the micropapillary growth pattern may not be exclusively associated with a worse prognosis or a distinct biology.^81^ In addition, data do not support any specific threshold of micropapillary morphology that is clinically significant in mucin‐producing cancers. As it is not certain that there is a distinct micropapillary subtype of MC, it is advised that a prominent micropapillary pattern in a MC can be noted in the pathology report but it is not designated as a specific subtype.^82^

Since pure MC is classically ER positive and HER2 negative and associated with a favourable prognosis, it is recommended that MCs that display high nuclear grade, ER negativity or HER2 positivity should be classified as IBC‐NST with mucin production rather than as MC. The importance of excluding a metastasis to the breast from another primary site is emphasised.

### Rare and Salivary Gland‐Type Tumours

**Figure 16:**
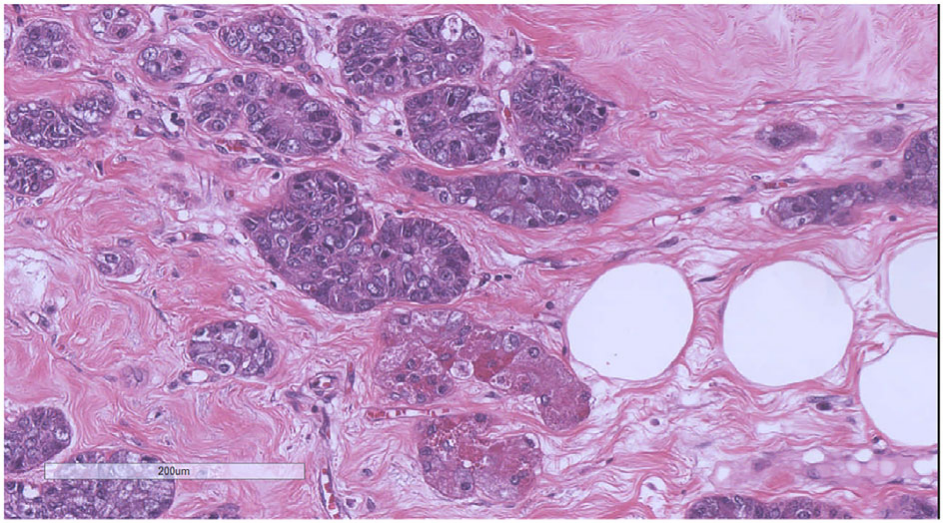
Acinic cell‐like carcinoma. The neoplastic cells are atypical and have granular cytoplasm. In some cells, coarse eosinophilic granules can be seen. [Colour figure can be viewed at wileyonlinelibrary.com]

Two changes have been introduced to the category of *rare and salivary gland‐type tumours*. Acinic cell carcinoma has been re‐named *acinic cell‐like carcinoma* (Figure 16) because, in contrast to acinic cell carcinomas of salivary glands, morphologically similar tumours occurring in the breast do not show the typical t(4;9)(q13;q31) rearrangement and/or NR4A3 protein overexpression.^83^ This rare breast tumour instead exhibits a molecular profile similar to that of conventional triple negative breast carcinoma and to carcinomas arising in association with microglandular adenosis.^83^, ^84^, ^85^, ^86^, ^87^, ^88^, ^89^, ^90^, ^91^, ^92^

*Polymorphous adenocarcinoma* has been removed from this 6th edition as there are only rare case reports and insufficient evidence to merit recognition as a distinct breast tumour type.

### Neuroendocrine Neoplasms

The application of neuroendocrine neoplasm (NEN) terminology continues to pose challenges for the classification of breast cancers in the 6th edition. Overlapping features with multiple histologic types, variability in neuroendocrine marker expression, and a lack of clinical treatment relevance in breast cancer have resulted in uncertainty and resistance to adoption of this terminology in practice.

In the 5th edition, the WHO Editorial Board aligned the classification of breast NENs with the 2018 expert consensus statement by the International Agency for Research on Cancer (IARC) and WHO,^93^ which proposed adopting the term ‘neuroendocrine neoplasm (NEN)’ to encompass all tumours with predominant neuroendocrine differentiation, including both well‐differentiated and poorly differentiated forms. Morphology and the expression of markers of neuroendocrine differentiation were recognised as key features defining these neoplasms at any specific anatomical site. Nevertheless, it was acknowledged that true NENs of the breast are rare and poorly defined. Apart from sporadic cases of small cell carcinoma, analogous to its pulmonary counterpart, the definition of NENs in the breast varied widely, resulting in variable incidence ranging from <0.1% to as high as 20%^94^, ^95^, ^96^ depending on the different diagnostic criteria applied. There is also overlap between NENs and breast carcinomas exhibiting NE differentiation such as IBC‐NST, solid papillary carcinoma, and hypercellular MC, which are regarded as distinct breast tumour entities, and should not be classified as NET or neuroendocrine carcinoma (NEC).

Continued changes in classification approaches for primary breast NE‐differentiated cancers have puzzled pathologists and created confusion in the clinical management of these patients.^97^, ^98^

The Editorial Board of the 6th edition of the WHO classification of breast tumours agreed that the 2018 proposed unified classification of NEN is difficult to apply to the breast. Apart from solid papillary carcinoma and hypercellular MC, which are distinct special type breast cancers, as well as the rarely encountered small cell carcinoma, it is uncertain if true primary NENs exist in the breast.^99^ Studies evaluating prognostic relevance of NE differentiation in invasive breast carcinoma have yielded variable results, which implies a need for refinement of the 2018 proposed unified classification of NEN.^100^ Currently, there is no conclusive clinical relevance to the presence of NE differentiation in invasive breast cancer, and routine staining of IBC‐NST for NE markers is not recommended.

**Figure 17:**
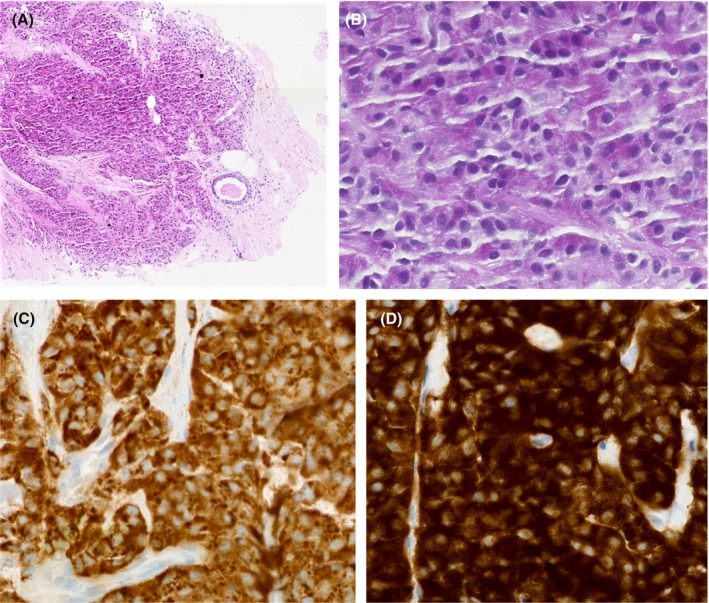
Metastatic NET to the breast (gastrointestinal primary). (**A**) Cellular tumour composed of nests and sheets of cells with hyperchromatic nuclei and granular cytoplasm (**B**). (**C**) Chromogranin and (**D**) Synaptophysin show diffuse expression indicating neuroendocrine differentiation. [Colour figure can be viewed at wileyonlinelibrary.com]

When a breast tumour with distinct NE morphology resembling a NET (previously referred to as ‘carcinoid tumour’, a term that is not recommended in the 6th edition) and which is histologically different from solid papillary carcinoma and MC manifests NE differentiation supported by adjunctive IHC, metastasis from another primary site such as the GI tract (Figure 17) and lung needs to be ruled out.^100^, ^101^

**Figure 18:**
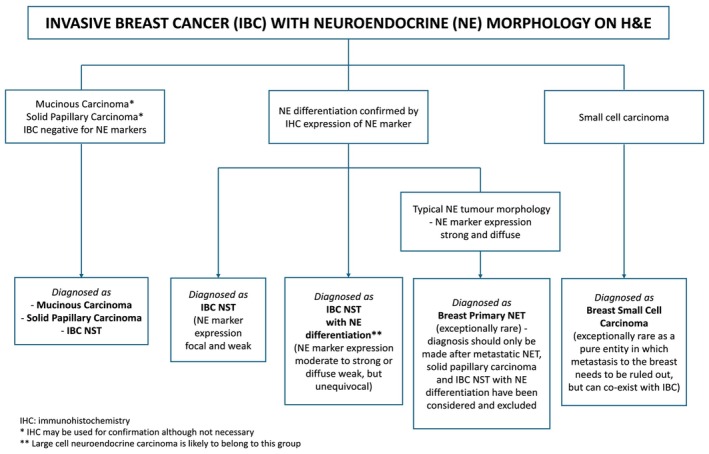
Diagnostic algorithm for invasive breast carcinoma with neuroendocrine morphology. [Colour figure can be viewed at wileyonlinelibrary.com]

A recommended algorithm for a diagnostic approach towards breast tumours displaying NE morphology, endorsed by the 6th edition Editorial Board, is shown in Figure 18.

Although assessment of NE differentiation is not recommended in routine practice, if performed, Figure 1 provides useful classification recommendations. This algorithm recognises that invasive breast cancers with neuroendocrine morphology exist. Among these, solid papillary carcinoma and hypercellular MC are special tumour types described in their respective sections in the 6th edition. Invasive carcinomas with morphological features highly suggestive of NE differentiation, but which are negative or demonstrate focal and weak NE marker expression, are classified as ‘IBC‐NST’.

Tumours in which NE marker expression is moderate to strong, or diffuse and weak but unequivocal, are classified as IBC‐NST with NE differentiation. ‘Large cell neuroendocrine carcinoma’ is likely to belong to this group of tumours but is not recognised as a separate entity with distinct clinical relevance. These tumours should be classified as IBC‐NST with NE differentiation and are included as a morphological pattern of invasive breast carcinoma NST in the 6th edition.

**Figure 19:**
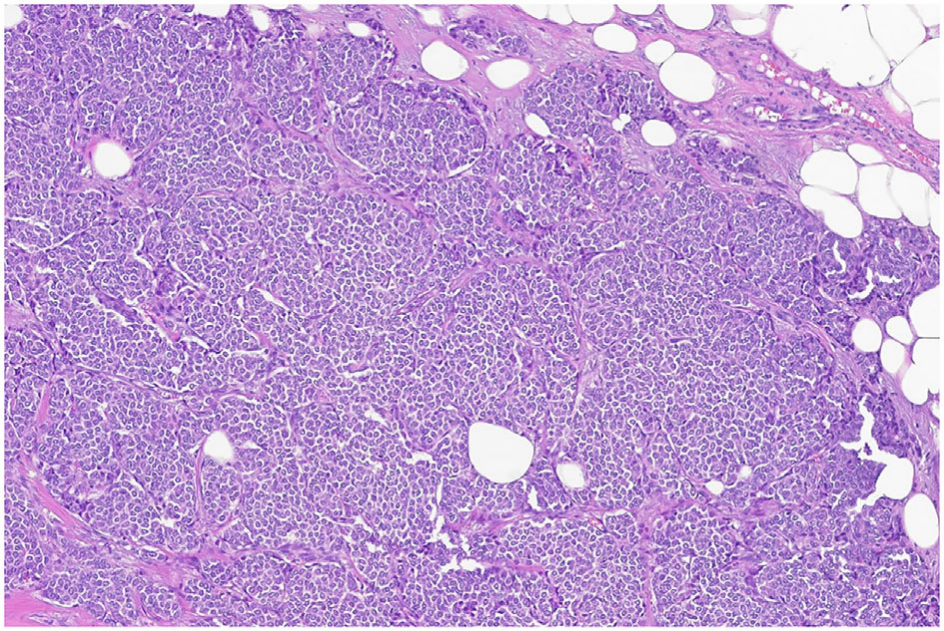
Neuroendocrine Tumour (NET) of the breast, grade 1. Solid islets of neoplastic cells, separated by collagen fibres, infiltrating fat lobules. [Colour figure can be viewed at wileyonlinelibrary.com]

Primary NETs of the breast are exceptionally rare and can be diagnosed only if the tumour displays the typical morphology of a NET (Figure 19) similar to other anatomical sites as well as strong, diffuse expression of NE markers. It is essential that metastatic NET, solid papillary carcinoma and invasive carcinoma NST with NE differentiation are primarily excluded.

Primary small cell neuroendocrine carcinoma (SCNEC) is regarded as a specific cytomorphological growth pattern of invasive breast cancers with neuroendocrine morphology, provided that metastasis to the breast has been ruled out. Primary SCNEC is exceptionally rare in its pure form, but it can co‐exist with IBC.

In terms of clinical treatment relevance, identifying neuroendocrine differentiation in a breast cancer usually does not alter treatment. Even the very rare SCNEC of the breast may be managed similarly to IBC‐NST in the primary setting. In patients with advanced or metastatic disease, treatment regimens similar to those used in small cell carcinoma of the lung may be attempted (data are very limited on effectiveness given its rarity).

## Fibroepithelial Tumours and Hamartomas of the Breast

### Phyllodes Tumours

A major update in the 6th edition chapter of breast phyllodes tumours is the revision of criteria for the diagnosis of malignancy.

In the 4th and 5th editions, in the absence of malignant heterologous elements, the diagnosis of malignant phyllodes tumours necessitated the presence of all five of the following features: marked stromal nuclear pleomorphism; stromal overgrowth defined by the absence of epithelial elements in one low‐power microscopic field (40× magnification: 4× objective and 10× eyepiece) containing only stroma; increased mitoses (≥5 mitoses/mm^2^; ≥10 mitoses per 10 high power fields of 0.5 mm in diameter and 0.2 mm^2^ in area); increased stromal cellularity which is marked and usually diffuse; and an infiltrative border. This approach aimed to avoid overdiagnosis of malignancy and improve diagnostic consistency. Emerging evidence, however, suggests that the requirement of all five of these adverse histological criteria can result in underdiagnosis of tumours with metastatic potential. Recent studies have highlighted cases where tumours classified as borderline phyllodes tumours by WHO criteria exhibited metastatic behaviour, emphasising the need to refine the diagnostic framework. In two studies with 140 malignant phyllodes tumours,^102^, ^103^ rare tumours developed distant metastasis even with 2 or 3 of the above features, although it is unclear whether these tumours were comprehensively sampled, which may have resulted in other adverse morphologic features not being identified.

**Figure 20:**
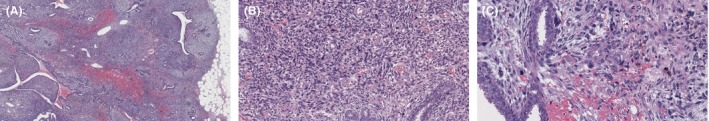
Malignant phyllodes tumour (case courtesy of Dr Mihir Gudi and Dr Sung Hock Chew). (**A**) Malignant phyllodes tumour shows four of five adverse histological criteria, with marked stromal hypercellularity, marked stromal atypia, mitoses exceeding 10 per 10 high power fields, irregular tumour borders, without stromal overgrowth. (**B**) Marked stromal hypercellularity is seen among benign ducts. (**C**) Marked stromal atypia and scattered stromal mitoses are present. [Colour figure can be viewed at wileyonlinelibrary.com]

An international group of breast pathologists with expertise and experience in diagnosing phyllodes tumours has proposed that malignant phyllodes tumours can be diagnosed when at least four of the five WHO adverse criteria are present, rather than requiring the ‘full house’ set of adverse histological parameters, supplemented by comprehensive tumour sampling and clinical context. This view balances the risk of underdiagnosis with the need for standardised, reproducible diagnostic practice for appropriate respective treatments.^104^ In light of recent evidence, the 6th edition WHO editorial board accepted that 4 adverse criteria are sufficient for the diagnosis of malignant phyllodes tumour (Figure 20), emphasising the importance of careful histological documentation, specifically the criteria used to support the malignant grade.

A pragmatic approach to the diagnosis of ‘grey zone’ phyllodes tumours showing histological features which straddle benign/borderline or borderline/malignant grades is provided in the 6th edition, pending more studies for morphology‐based algorithms which predict clinical behaviour.^105^ In a well sampled phyllodes tumour with histological features in both benign and borderline grades, the presence of more features in the benign category with slight deviation into borderline criteria [for example mitoses that exceed the usual number of <5 mitoses per 10 high power fields (<2.5 mitoses per mm^2^); or focal border irregularity], the case may be regarded as favouring a benign phyllodes tumour with a comment on which histological parameters are outside the benign category. If most features belong to the borderline rather than benign category, the case should be assigned as borderline. In the scenario of overlapping features between borderline and malignant phyllodes tumours, if features are predominantly in the borderline category with occasional histological parameters (less than 4 adverse criteria) at the malignant end, the case may be deemed borderline with explanatory comments (Table 1).

**Table 1:** Summary of the histological features of fibroadenoma and phyllodes tumours, incorporating WHO 6th edition updates

Histological feature	Fibroadenoma	Phyllodes tumours		
		Benign	Borderline	Malignant*
Tumour border	Well defined^†^	Well defined^**‡**^	Well defined, may be focally permeative	Permeative
Stromal cellularity	Variable, scant to uncommonly cellular,^§^ usually uniform	Cellular, usually mild, may be non‐uniform or diffuse	Cellular, usually moderate, may be non‐uniform or diffuse	Cellular, usually marked and diffuse
Stromal atypia	None	Mild or none	Mild or moderate	Marked
Mitotic activity	Usually none, rarely low^§^	Usually low: <2.5 mitoses/mm^2^ (<5 mitoses/10 HPF)	Usually frequent: 2.5 to <5 mitoses/mm^2^ (5–9 mitoses/10 HPF)	Usually abundant: ≥5 mitoses/mm^2^ (≥10 mitoses/10 HPF)
Stromal overgrowth	Absent	Absent	Absent (or very focal)	Often present
Malignant heterologous elements	Absent	Absent	Absent	May be present^¶^
Distribution relative to all breast tumours	Common	Uncommon	Rare	Rare
Relative proportion of all phyllodes tumours	Not applicable	60%–75%	15%–26%	8%–20%

* Diagnosis of malignant phyllodes tumours requires the presence of at least four of the five histological features of permeative tumour borders, marked and diffuse stromal cellularity, marked stromal atypia, ≥10 stromal mitoses/10 HPF, and stromal overgrowth.

^†^ Occasional typical fibroadenomas may have a slightly irregular interphase with adjacent stroma.

^‡^ Occasional benign phyllodes tumours may possess slightly irregular borders, though other features are of the benign grade.

^§^ Fibroadenomas in young people may have increased stromal cellularity and increased mitoses.

^¶^ The presence of malignant heterologous elements (except well‐differentiated liposarcoma) in a phyllodes tumour, in the absence of other adverse histological features, allows a malignant categorisation.

Appropriate sampling protocols are essential for accurate grading of phyllodes tumours. In large tumours or those in which there are features suggesting malignancy, extensive sampling with at least 2 blocks per 1 cm of the largest tumour dimension is recommended to avoid missing the malignant component, which can be very focal due to inherent heterogeneity.^106^

## Other changes

Mesenchymal and haematological tumours included in this edition have been restricted to those occurring more commonly or principally in the breast. The mesenchymal tumours chapter focuses on fibromatosis, myofibroblastoma, granular cell tumour, pseudoangiomatous stromal hyperplasia (PASH) as well as various vascular lesions (categorised based on the ISSVA classification)^107^, ^108^, ^109^ that occur in the breast. For discussion of entities that may be seen in the breast but occurring more commonly at other anatomic sites, the reader is referred to the WHO Classification of Tumours, Soft Tissue and Bone Volume.^110^ Using a similar approach, the haematolymphoid tumours chapter was restricted to tumour types that may arise specifically in the breast, such as breast implant‐associated anaplastic large cell lymphoma, or have occasional tropism to the breast parenchyma, such as extranodal marginal zone lymphoma. For a detailed discussion of other haematolymphoid tumours that may involve the breast, the reader is referred to the WHO Classification of Haematolymphoid Tumours Volume.^107^

The Genetic tumour syndromes chapter has also been updated. Terminology is aligned with the WHO Classification of Tumours and Genetic Tumour syndromes volume to facilitate standardisation and clear communication.^111^

## Conclusion

The classification of breast tumours continues to evolve in accordance with emerging evidence that guides our approach to their diagnosis and categorisation, with the ultimate global goal of accurate prognostication for optimal patient management across diverse geographical regions. With the vast amounts of literature accumulating during periods between the WHO editions, the WCT EVI Map project helmed by WHO‐IARC serves to provide a living evidence gap map that informs the quality and strength of evidence that can be prioritised in updating and revising the classification of these tumours.

In this summary, we highlight key updates of the 6th edition of the WHO classification of breast tumours. It is hoped that enthusiastic research will be further encouraged in uncommon tumour types where evidence remains limited.

## Author Contributions

CQ and PHT wrote the manuscript. All authors read, provided input and approved the final manuscript.

## Funding Information

The authors received no specific funding for this work. EB is supported, in part, by a Cancer Centre Support Grant of the National Institutes of Health/National Cancer Institute [Grant no. P30CA0087478].

## Conflict of Interest

ML reports advisory role for Roche, Lilly, Novartis, AstraZeneca, Pfizer, Gilead, MSD, Pierre Fabre, Menarini, Nordic Pharma, Ipsen, Daiichi Sankyo and Bayer; receiving speaker honoraria from Roche, Lilly, Novartis, Pfizer, Sandoz, Libbs, Daiichi Sankyo, Takeda, Ipsen, Menarini and AstraZeneca; receiving travel grants from Gilead, Roche and Daiichi Sankyo; receiving research funding (to his institution) from Gilead, all outside the present work. RS declares Advisory Board role for BMS, Roche, Exact Sciences, Daicchii Sankyo, Astra Zeneca. Research funding by Roche, Puma, Merck, BMS and travel and congress‐registration support by Roche, Merck, Case45 and Astra Zeneca. SF reports advisory roles for Novartis, BMS, MSD, AZ, AbbVie, Roche, Amgen, Menarini, Janssen, Boehringer Ingelheim and Bayer. He is in receipt of a National Health and Medical Research Council grant (2020/GNT1193630). The other authors declare no relevant competing interests.

## IARC/WHO Disclaimer

The content of this article represents the personal views of the authors and does not represent the views of the authors' employers and associated institutions. Where authors are identified as personnel of the International Agency for Research on Cancer/World Health Organization, the authors alone are responsible for the views expressed in this article and they do not necessarily represent the decisions, policy, or views of the International Agency for Research on Cancer/World Health Organization.

## WCT Standing Editorial Board

Wendy Cooper, Department of Tissue Pathology and Diagnostic Oncology, Royal Prince Alfred Hospital, NSW Health Pathology, Camperdown, Australia. Michael Eden, Department of Pathology, Cambridge University Hospitals NHS Foundation Trust, Cambridge, United Kingdom. Andrew Field, Department of Anatomical Pathology, St Vincent's Hospital, Sydney, and University of NSW and University of Notre Dame, Darlinghurst, Sydney, NSW, Australia. Vicky Goh, Department of Radiology, King's College London and Guy's and St Thomas' Hospitals, London, United Kingdom. Jennelle C. Hodge, Department of Medical and Molecular Genetics, and IU Simon Comprehensive Cancer Center, Indiana University, Indianapolis, USA. James Kench, Department of Tissue Pathology & Diagnostic Oncology, Royal Prince Alfred Hospital, Camperdown, Sydney, Australia. Joseph D. Khoury, Department of Pathology, Microbiology, and Immunology, University of Nebraska Medical Center, Omaha, USA. Katia Leite, Department of Urology, University of Sao Paulo, Sao Paulo, Brazil. Zhiyong Liang, Department of Pathology, Peking Union Medical College Hospital, Beijing, China. Daichi Maeda, Department of Molecular and Cellular Pathology, Kanazawa University, Kanazawa, Japan. George Netto, Department of Pathology & Laboratory Medicine, Hospital of the University of Pennsylvania, Philadelphia, USA. Bharat Rekhi, Department of Pathology, Tata Memorial Centre, Mumbai, India. Miguel Reyes Mugica, Department of Pathology and Laboratory Medicine, University of Miami Miller School of Medicine, Miami, USA. Brian Rous, Department of Histopathology, NHS England, Cambridge, United Kingdom. Ales Ryska, The Fingerland Department of Pathology, University Hospital Hradec Kralove, Czechia. Shahin Sayed, Department of Pathology, Aga Khan University Hospital, Nairobi, Kenya. Antonia Sepulveda, Department of Pathology, George Washington University School of Medicine and Health Sciences, Washington, DC, USA. Chanjuan Shi, Department of Pathology, Duke University Medical Center, Durham, USA. Gary Tse, Department of Anatomical and Cellular Pathology, The Chinese University of Hong Kong, Hong Kong SAR China.

## Data Availability

Data sharing not applicable to this article as no data sets were generated or analysed during the current study.
